# Supplementary material for: tRNA-derived RNA processing in sperm transmits non-genetically inherited phenotypes to offspring in C. elegans
Source: Nat Commun. 2026 Mar 15;17:3999. doi: 10.1038/s41467-026-70029-7 (PMC13136342; doi:10.1038/s41467-026-70029-7)
Supplement: Supplementary file 1 — Supplementary Information [file 41467_2026_70029_MOESM1_ESM.pdf]

tRNA-derived RNA processing in sperm transmits non-genetically inherited phenotypes to offspring in *C. elegans*

Supplemental Information

Supplemental Methods

Northern blot

Probe sequence

tDR Gly-GCC 5’ AGGCGAGCATTCTACCACTGAACCACCGAT 3’

tDR Glu-CTC 5’AGCCATAAATCCTAACCACTAGACCACAACGGA 3’

U6 5’ TTGCGTGTCATCCTTGCGCAGG 3’

Supplemental Figures

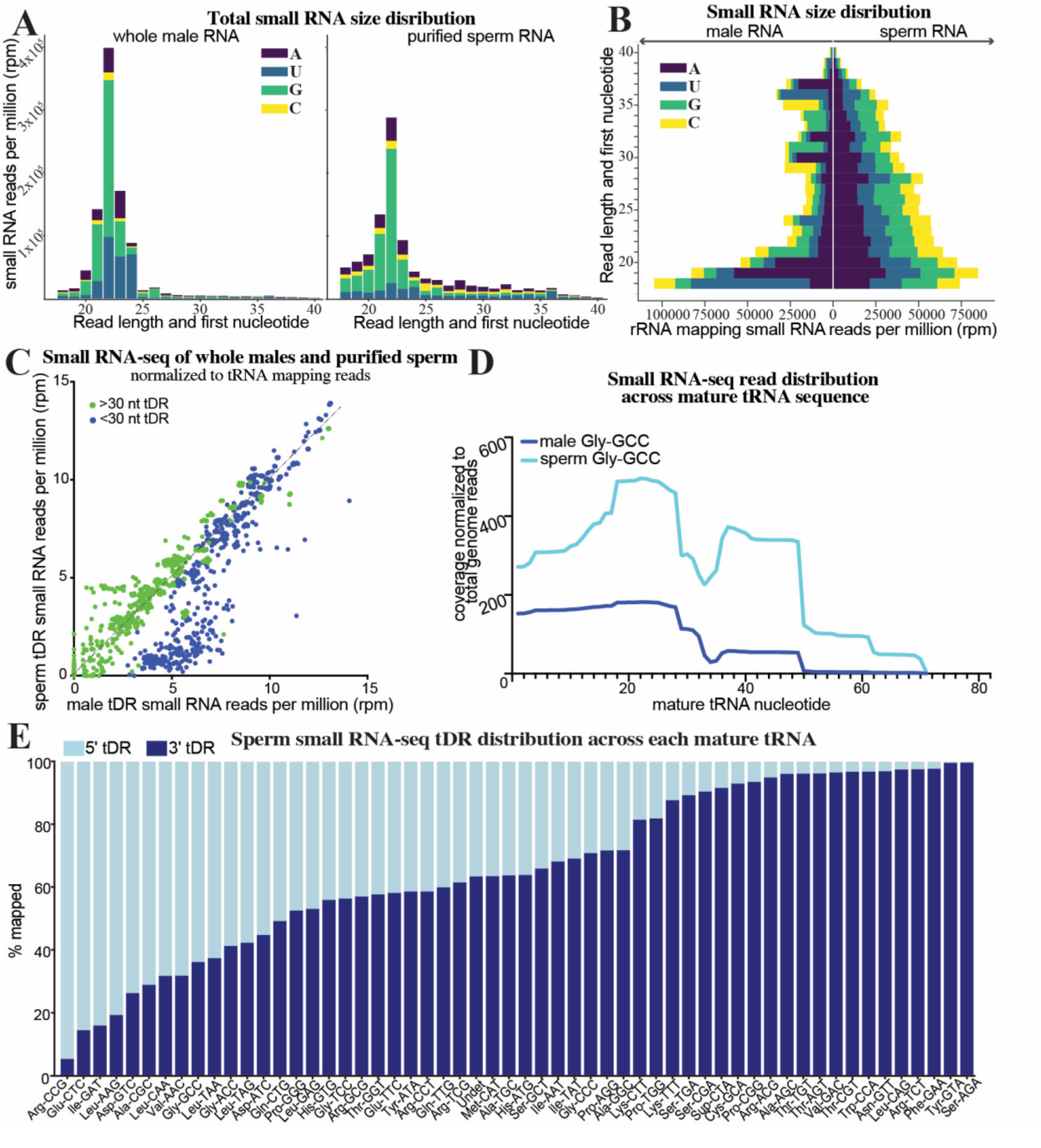

Supplemental Figure 1. Small RNA-seq reveals that tDRs are enriched in *C. elegans* sperm. Small RNA-seq was performed on PAGE purified 18-40 nt RNA from *C. elegans* whole males and purified sperm. **(A)** Length (x-axis) and first nucleotide distribution (legend) of all genome mapping reads. Whole male data (left) and purified sperm (right). **(B)** Reads mapping to rRNAs were quantitated for RNA length (y-axis) and plotted as their starting nucleotide in a diverging bar-graph (x-axis – total quantitated reads). **(C)** tRNA mapping reads were separated as >30 or <30 nucleotides in length and quantitated, as reads per million (rpm) tRNA mapping reads, for all

tRNA isoacceptor genes. Each dot represents a quantitated tRNA isoacceptor (green >30 and blue <30 nucleotide tRNA mapping reads per tDR). **(D)** Reads mapping to tRNA Gly-GCC were quantitated as read depth (y-axis – number of times each nucleotides mapped) across the mature tRNA sequence (x axis**)** **(E)** Distribution of 5′- and 3′-derived tRNA fragments across individual tRNA isoacceptors from sperm small RNA-seq data. For each mature tRNA isoacceptor, the average fraction across biological replicates of mapped tDR reads originating from the 5′ arm (light) or 3′ arm (dark) is shown, calculated relative to total tDR reads mapping to that isoacceptor.

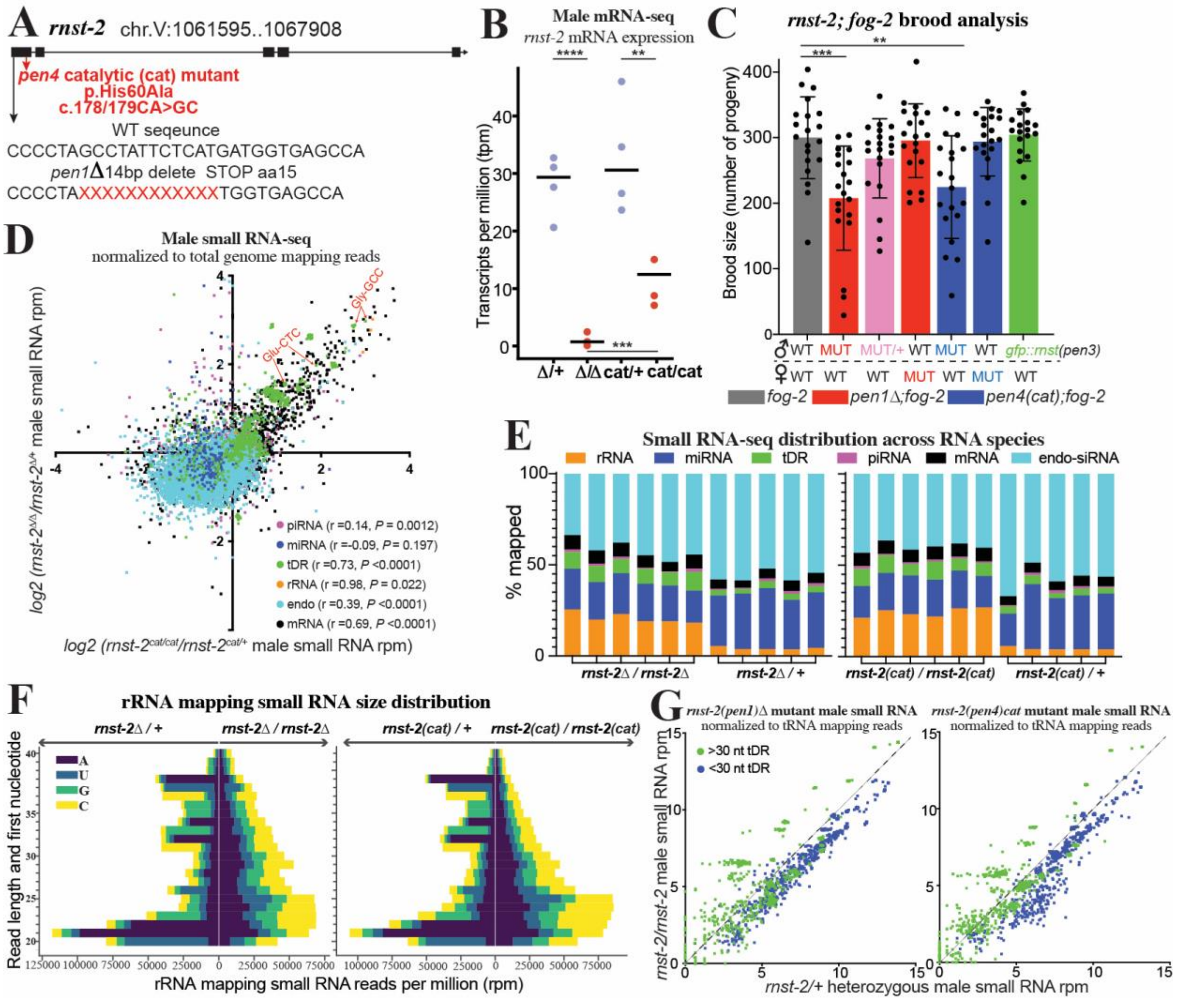

Supplemental Figure 2. *rnst-2* regulates tDR accumulation and length. Small RNA-seq was performed on *C. elegans* whole male *rnst-2* mutant (*pen1* – Δ and *pen4* – catalytic) as well as heterozygous controls. **(A)** Schematic of the *rnst-2* locus. Additionally, mutant alleles generated in this study in the proceeding experiments are annotated. **(B)** mRNA-seq was performed on whole male RNA from *rnst-2*$^{\Delta/\Delta}$, *rnst-2*$^{cat/cat}$, and heterozygous controls (*rnst-2*$^{\Delta/+}$ & *rnst-2*$^{cat/+}$) with the expression of *rnst-2* mRNA plotted for each sample (n = 3, ****P <0.0001 ***P <0.001 **P <0.01 unpaired students t-test). **(C)** Fecundity was measured by assessing the brood size (# of

progeny generated) of *rnst-2*$^{\Delta/\Delta}$, *rnst-2*$^{cat/cat}$, heterozygous controls (*rnst-2*$^{\Delta/+}$ & *rnst-2*$^{cat/+}$), endogenously(CRISPR) tagged *gfp::rnst-2*, and WT (*fog-2*) males mated with WT *fog-2* females (**$P_{<0.01}$, ***$P_{<0.001}$ – two-sample unpaired t-test). **(D)** The fold-change for each small RNA was plotted for *rnst-2*$^{\Delta/\Delta}$ versus *rnst-2*$^{\Delta\ +}$(x-axis) compared to *rnst-2*$^{cat/cat}$ versus *rnst-2*$^{cat/+}$(y-axis).**(E)** Total small RNA reads mapping to each class of RNAs quantitated as percentage of total genome mapping reads as stacked bar graphs for each replicate of the experiment (n = 4). Color legend is the same as in A. **(F)** Reads mapping to rRNAs were quantitated for RNA length (y-axis) and plotted as their starting nucleotide in a diverging bar-graph (x-axis – total quantitated reads). Read-length distributions were compared between conditions using two-sided Mann–Whitney U tests with Benjamini–Hochberg false discovery rate (FDR) correction; asterisks indicate bins with FDR-adjusted $P < 0.05$. **(G)** For each mutant and their respective heterozygous control, tRNA mapping reads were separated as >30 or <30 nucleotides in length and quantitated, as reads per million (rpm), for all tRNA isoacceptor genes. Each dot represents a quantitated tRNA isodecoder (green >30 and blue <30 nucleotide tRNA mapping reads for each isodecoder).

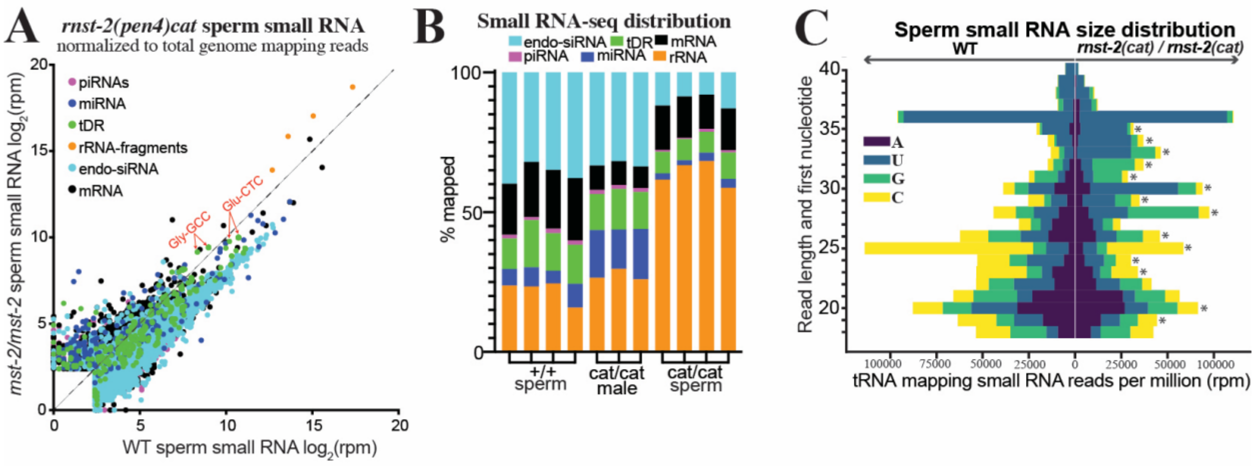

Supplemental Figure 3. *rnst-2* regulates tDR accumulation in sperm. Small RNA-seq was performed on *rnst-2(cat)* sperm and whole males and compared to WT sperm small RNA data. **(A)** Scatter plot of small RNA-seq data normalized to total genome mapping reads from WT sperm (x-axis – also Fig.1) and *rnst-2*$^{cat/cat}$ mutant sperm (y-axis). **(B)** Distribution of small RNAs mapped to all small RNA classes from WT and *rnst-2*$^{cat/cat}$ mutant sperm and males. Each biological replicate from the experiment is represented as an individual bar graph. **(C)** Reads mapping to tRNAs were quantitated for RNA length (y-axis) and plotted as their starting nucleotide (legend) in a diverging bar-graph (x-axis – total quantitated reads) for WT and *rnst-2*$^{cat/cat}$ mutant sperm. Read-length distributions were compared between conditions using two-sided Mann–Whitney U tests with Benjamini–Hochberg false discovery rate (FDR) correction; asterisks indicate bins with FDR-adjusted $P < 0.05$.

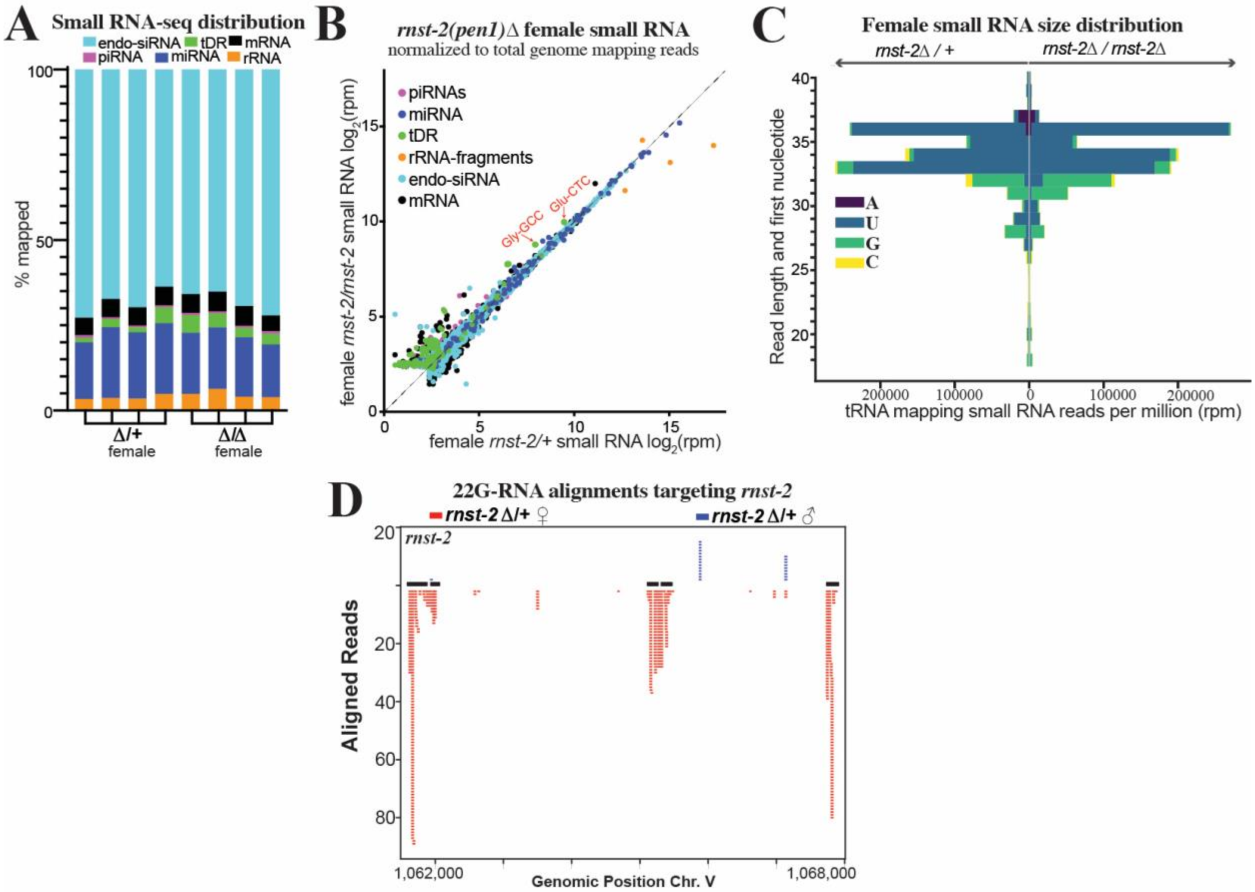

Supplemental Figure 4. ***rnst-2*** **dependent regulation of tDRs is suppressed in females.** Small RNA-seq was performed on *C. elegans* whole female (without embryos) *rnst-2*$^{\Delta/\Delta}$ mutants as well as heterozygous controls. **(A**) Distribution of small RNAs mapped to all small RNA classes from *rnst-2*$^{\Delta/+}$and *rnst-2*$^{\Delta/\Delta}$ females. Each biological replicate from the experiment is represented as an individual bar graph (n=4). **(B)** Scatter plot of small RNA-seq data normalized to total genome mapping reads as reads per million (rpm) from *rnst-2*$^{\Delta/+}$ (x-axis) and *rnst-2*$^{\Delta/\Delta}$ females (y-axis). **(C)** Reads mapping to tRNAs were quantitated for RNA length (y-axis) and plotted as their starting nucleotide (legend) in a diverging bar-graph (x-axis – total quantitated reads) for *rnst-2*$^{\Delta/+}$and *rnst-2*$^{\Delta/\Delta}$ females. Read-length distributions were compared between conditions using two-sided Mann–Whitney U tests with Benjamini–Hochberg false discovery rate (FDR) correction; asterisks indicate bins with FDR-adjusted $P < 0.05$. **(D)** 22G endo-siRNA reads aligned to the *rnst-2* gene locus in *rnst-2*$^{\Delta/+}$ male (Fig. 2) and female small RNA data.

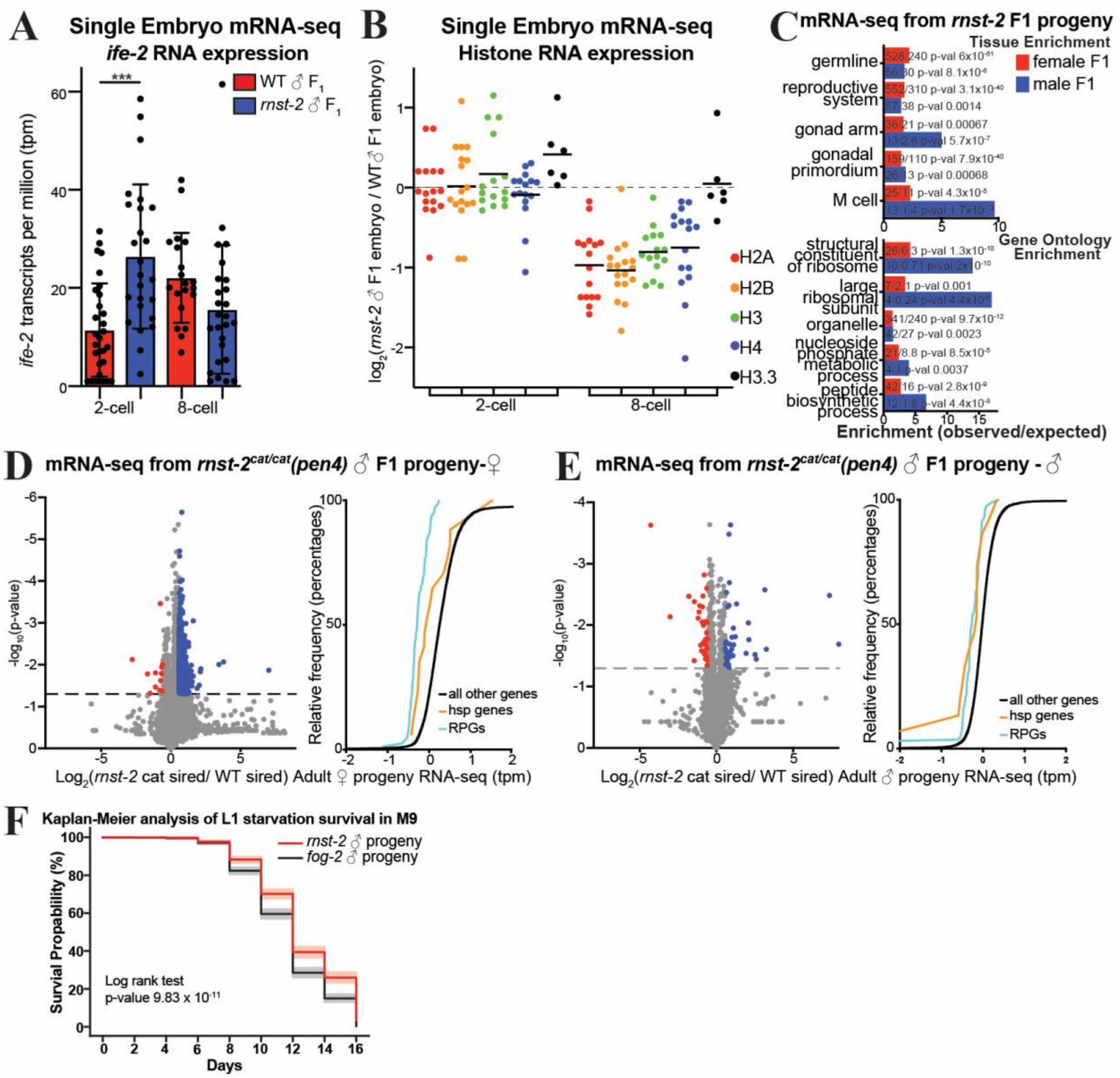

Supplemental Figure 5. ***rnst-2*** **mutant males transmit non-genetically inherited phenotypes to offspring. (A)** Bar-plot representing *ife-2* expression in transcripts per million (tpm) for each replicate in our dataset in 2- and 8-cell embryos sired by *rnst-2*$^{\Delta/\Delta}$ and WT males (***$P < 0.001$ – two-sample t-test); n = 20-30. **(B)** Expression of histone RNAs grouped by type of histone gene in 2- and 8-cell embryos sired by *rnst-2*$^{\Delta/\Delta}$ and WT males. Each dot represents the average expression of a specific histone gene in *rnst-2*$^{\Delta/\Delta}$ relative to WT sired progeny. **(C)** Significantly regulated Gene Ontology and Tissue Enrichment terms for genes differentially expressed in mRNA-seq data from male and female progeny of *rnst-2*$^{\Delta/\Delta}$ males. **(D-E)** Volcano plots graphing the mRNA-seq data of progeny of *rnst-2*$^{\Delta/\Delta}$ males compared to WT male progeny. Additionally, cumulative distribution frequency plots of gene expression of *rnst-2*$^{\Delta/\Delta}$ relative to control progeny for all genes, heat-shock genes, and ribosomal protein genes (RPGs). **(D)** Female progeny mRNA-seq (RPGs P <0.0001 Kolmogorov-Smirnov test – hsp genes $P < 0.05$) **(E)** Male progeny mRNA-seq (RPGs P <0.0001– hsp genes $P < 0.01$. **(F)** The L1 progeny of *rnst-2*$^{\Delta/\Delta}$ or WT *fog-2* males mated with WT females were assessed for starvation survival over a time course in M9 medium. Five independent

experiments were performed, and survival curves were generated using the Kaplan-Meier method with the lifelines package in Python. Statistical differences (P value) were calculated using the log-rank test to compare survival distributions over time, with the grey and pink shaded areas representing 95% confidence intervals.

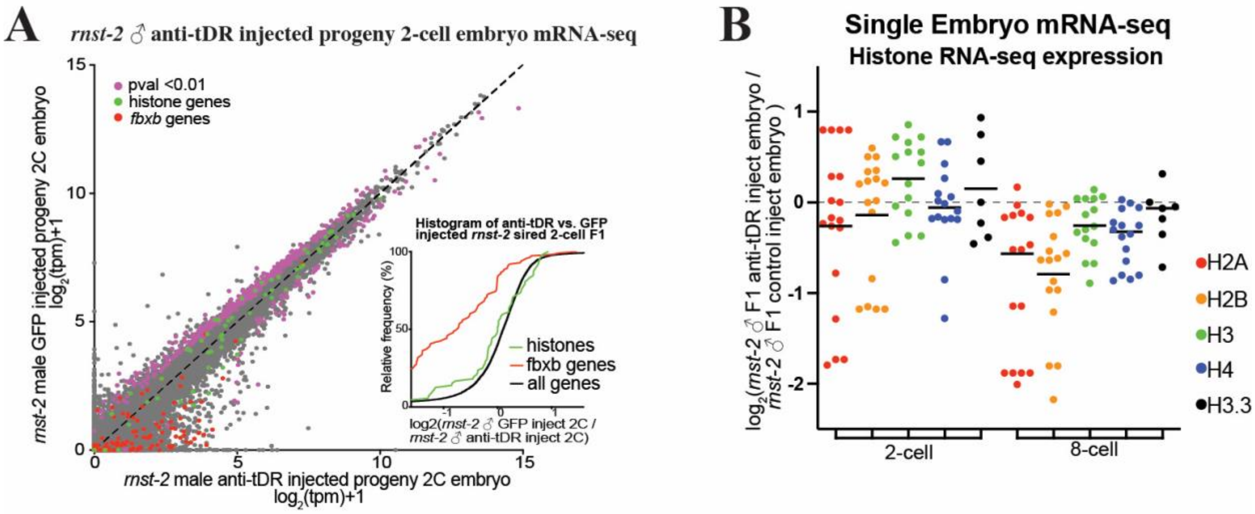

Supplemental Figure 6. Specific tDRs contribute to the transmission non-genetically inherited phenotypes to progeny. (A-B) Single-embryo mRNA-seq was performed on 2-cell and 8-cell embryos sired by *rnst-2*$^{\Delta/\Delta}$ males mated with WT females and then microinjected with either anti-tDR (anti-Gly-GCC or anti-Glu-CTC) + GFP mRNA or GFP mRNA alone as a microinjection control. **(A)** tpm normalized mRNA-seq data represented as a scatter plot of 2-cell embryo progeny of *rnst-2*$^{\Delta/\Delta}$ males mated with WT females injected with anti-tDR (x-axis) versus 2-cell embryo progeny of *rnst-2*$^{\Delta/\Delta}$ mated with WT female injected with GFP mRNA (y-axis). The inset graph represents cumulative distribution frequency plots of all genes, histone genes (colored green), and *fbxb* genes (colored red). n = 26 GFP injected, n = 23 anti-tDR injected **(B)** Expression of histone RNAs grouped by type of histone gene in 2 and 8-cell embryos sired by *rnst-2*$^{\Delta/\Delta}$ males with WT females microinjected with either anti-tDR or GFP control RNA. Each dot represents the average of a specific histone gene's expression in *rnst-2*$^{\Delta/\Delta}$ relative to WT sired progeny.
