# Supplementary material for: tRNA-derived RNA processing in sperm transmits non-genetically inherited phenotypes to offspring in C. elegans
Source: Nat Commun. 2026 Mar 15;17:3999. doi: 10.1038/s41467-026-70029-7 (PMC13136342; doi:10.1038/s41467-026-70029-7)
Supplement: Supplementary file 2 — Reporting Summary [file 41467_2026_70029_MOESM2_ESM.pdf]

nature portfolio

Corresponding author(s): Colin Conine

Last updated by author(s): Dec 23, 2025

Reporting Summary

Nature Portfolio wishes to improve the reproducibility of the work that we publish. This form provides structure for consistency and transparency in reporting. For further information on Nature Portfolio policies, see our Editorial Policies and the Editorial Policy Checklist.

Statistics

For all statistical analyses, confirm that the following items are present in the figure legend, table legend, main text, or Methods section.

n/a	Confirmed	
☐	☒	The exact sample size (n) for each experimental group/condition, given as a discrete number and unit of measurement
☐	☒	A statement on whether measurements were taken from distinct samples or whether the same sample was measured repeatedly
☐	☒	The statistical test(s) used AND whether they are one- or two-sided<br>*Only common tests should be described solely by name; describe more complex techniques in the Methods section.*
☐	☒	A description of all covariates tested
☐	☒	A description of any assumptions or corrections, such as tests of normality and adjustment for multiple comparisons
☐	☒	A full description of the statistical parameters including central tendency (e.g. means) or other basic estimates (e.g. regression coefficient) AND variation (e.g. standard deviation) or associated estimates of uncertainty (e.g. confidence intervals)
☐	☒	For null hypothesis testing, the test statistic (e.g. F, t, r) with confidence intervals, effect sizes, degrees of freedom and P value noted<br>*Give P values as exact values whenever suitable.*
☐	☒	For Bayesian analysis, information on the choice of priors and Markov chain Monte Carlo settings
☐	☒	For hierarchical and complex designs, identification of the appropriate level for tests and full reporting of outcomes
☐	☒	Estimates of effect sizes (e.g. Cohen's d, Pearson's r), indicating how they were calculated

Our web collection on statistics for biologists contains articles on many of the points above.

Software and code

Policy information about availability of computer code

Data collection	No custom software was used for data collection
Data analysis	GraphPad Prism 10,ImageJ 1.54, and Python 3.9 were used to generate graphs an for statistical analysis.

For manuscripts utilizing custom algorithms or software that are central to the research but not yet described in published literature, software must be made available to editors and reviewers. We strongly encourage code deposition in a community repository (e.g. GitHub). See the Nature Portfolio guidelines for submitting code & software for further information.

Data

Policy information about availability of data

All manuscripts must include a data availability statement. This statement should provide the following information, where applicable:

- Accession codes, unique identifiers, or web links for publicly available datasets
- A description of any restrictions on data availability
- For clinical datasets or third party data, please ensure that the statement adheres to our policy

Data Availability
All RNA sequencing data can be accessed via GEO, with the accession number GSE293020. Additionally, all generated data is provided in the Source Data file.

Research involving human participants, their data, or biological material

Policy information about studies with human participants or human data. See also policy information about sex, gender (identity/presentation), and sexual orientation and race, ethnicity and racism.

Reporting on sex and gender	*Use the terms sex (biological attribute) and gender (shaped by social and cultural circumstances) carefully in order to avoid confusing both terms. Indicate if findings apply to only one sex or gender; describe whether sex and gender were considered in study design; whether sex and/or gender was determined based on self-reporting or assigned and methods used.*<br>*Provide in the source data disaggregated sex and gender data, where this information has been collected, and if consent has been obtained for sharing of individual-level data; provide overall numbers in this Reporting Summary. Please state if this information has not been collected.*<br>*Report sex- and gender-based analyses where performed, justify reasons for lack of sex- and gender-based analysis.*
Reporting on race, ethnicity, or other socially relevant groupings	*Please specify the socially constructed or socially relevant categorization variable(s) used in your manuscript and explain why they were used. Please note that such variables should not be used as proxies for other socially constructed/relevant variables (for example, race or ethnicity should not be used as a proxy for socioeconomic status).*<br>*Provide clear definitions of the relevant terms used, how they were provided (by the participants/respondents, the researchers, or third parties), and the method(s) used to classify people into the different categories (e.g. self-report, census or administrative data, social media data, etc.)*<br>*Please provide details about how you controlled for confounding variables in your analyses.*
Population characteristics	*Describe the covariate-relevant population characteristics of the human research participants (e.g. age, genotypic information, past and current diagnosis and treatment categories). If you filled out the behavioural & social sciences study design questions and have nothing to add here, write "See above."*
Recruitment	*Describe how participants were recruited. Outline any potential self-selection bias or other biases that may be present and how these are likely to impact results.*
Ethics oversight	*Identify the organization(s) that approved the study protocol.*

Note that full information on the approval of the study protocol must also be provided in the manuscript.

Field-specific reporting

Please select the one below that is the best fit for your research. If you are not sure, read the appropriate sections before making your selection.

[x] Life sciences [ ] Behavioural & social sciences [ ] Ecological, evolutionary & environmental sciences

For a reference copy of the document with all sections, see nature.com/documents/nr-reporting-summary-flat.pdf

Life sciences study design

All studies must disclose on these points even when the disclosure is negative.

Sample size	As is standards in most C. elegans research 3+ biological replicates for both experimental and control were used for all experiments. Sample sizes were not predetermined using formal power calculations. Sample sizes were selected based on prior literature, established practices in the field, and pilot experiments that demonstrated reproducible effect sizes. Experiments were independently replicated, and biological replicates were used throughout. Sample sizes were sufficient to detect statistically significant differences using appropriate statistical tests, as reported in the figure legends.
Data exclusions	No data was excluded.
Replication	All experiment were biologically replicated at least 3 times. All attempts at replication of results were successful.
Randomization	Randomization is not relevant to this study. Samples and organisms were allocated to experimental groups based on genotype or experimental treatment rather than randomization. Covariates were controlled by using a common genetic background, age-matched organisms, and identical handling and experimental conditions.
Blinding	For phenotypic studies on rnst-2 offspring phenotypes groups were experimental and control groups were blindly assessed. For all other experiments Investigators were not blinded to group allocation. Blinding was not feasible or not relevant, as experimental groups were defined by genotype or molecular perturbation and outcomes consisted of quantitative molecular measurements analyzed using objective computational pipelines, minimizing potential observer bias.

Reporting for specific materials, systems and methods

We require information from authors about some types of materials, experimental systems and methods used in many studies. Here, indicate whether each material, system or method listed is relevant to your study. If you are not sure if a list item applies to your research, read the appropriate section before selecting a response.

Materials & experimental systems

n/a	Involved in the study	
☐	☒	Antibodies
☒	☐	Eukaryotic cell lines
☒	☐	Palaeontology and archaeology
☐	☒	Animals and other organisms
☒	☐	Clinical data
☒	☐	Dual use research of concern
☒	☐	Plants

Methods

n/a	Involved in the study	
☒	☐	ChIP-seq
☒	☐	Flow cytometry
☒	☐	MRI-based neuroimaging

Antibodies

Antibodies used

anti-digoxigenin-AP, Fab fragments (Roche, Cat No 11214667001)

Validation

Northern blotting using a 1:1000 dilution of anti-digoxigenin-AP, Fab fragments (Roche, Cat No 1110932774910- lot# 65752122) antibody detecting digoxigenin-labeled DNA oligonucleotide probes as described in the below citation.

Scacchetti A, Shields EJ, Trigg NA, Lee GS, Wilusz JE, Conine CC, Bonasio R. A ligation-independent sequencing method reveals tRNA-derived RNAs with blocked 3' termini. Mol Cell. 2024 Oct 3;84(19):3843-3859.e8. doi: 10.1016/j.molcel.2024.07.008. Epub 2024 Aug 2. PMID: 39096899; PMCID: PMC11455606.

Animals and other research organisms

Policy information about studies involving animals; ARRIVE guidelines recommended for reporting animal research, and Sex and Gender in Research

Laboratory animals

C. elegans

Wild animals

This study did not involve wild animals

Reporting on sex

When appropriate C. elegans male and females were separately isolated for analyses.

Field-collected samples

This study did not involved animals collected from the field.

Ethics oversight

All research performed in this study complies with all relevant ethical regulations. This research was approved by the University of Pennsylvania Institutional Biosafety Committee (IBC#24-315).

Note that full information on the approval of the study protocol must also be provided in the manuscript.

Plants

Seed stocks

Report on the source of all seed stocks or other plant material used. If applicable, state the seed stock centre and catalogue number. If plant specimens were collected from the field, describe the collection location, date and sampling procedures.

Novel plant genotypes

Describe the methods by which all novel plant genotypes were produced. This includes those generated by transgenic approaches, gene editing, chemical/radiation-based mutagenesis and hybridization. For transgenic lines, describe the transformation method, the number of independent lines analyzed and the generation upon which experiments were performed. For gene-edited lines, describe the editor used, the endogenous sequence targeted for editing, the targeting guide RNA sequence (if applicable) and how the editor was applied.

Authentication

Describe any authentication procedures for each seed stock used or novel genotype generated. Describe any experiments used to assess the effect of a mutation and, where applicable, how potential secondary effects (e.g. second site T-DNA insertions, mosiacism, off-target gene editing) were examined.
